# Competition between Ferroelectric and Ferroelastic Domain Wall Dynamics during Local Switching in Rhombohedral PMN-PT Single Crystals

**DOI:** 10.3390/nano12213912

**Published:** 2022-11-06

**Authors:** Denis Alikin, Anton Turygin, Andrei Ushakov, Mikhail Kosobokov, Yurij Alikin, Qingyuan Hu, Xin Liu, Zhuo Xu, Xiaoyong Wei, Vladimir Shur

**Affiliations:** 1 School of Natural Sciences and Mathematics, Ural Federal University, 620000 Ekaterinburg, Russia; 2 Electronic Materials Research Laboratory, Key Laboratory of the Ministry of Education & International Center for Dielectric Research, Xi’an Jiaotong University, Xi’an 710049, China

**Keywords:** ferroelectric domain structure, polarization reversal, crystal anisotropy, piezoresponse force microscopy

## Abstract

The possibility to control the charge, type, and density of domain walls allows properties of ferroelectric materials to be selectively enhanced or reduced. In ferroelectric–ferroelastic materials, two types of domain walls are possible: pure ferroelectric and ferroelastic–ferroelectric. In this paper, we demonstrated a strategy to control the selective ferroelectric or ferroelastic domain wall formation in the (111) single-domain rhombohedral PMN-PT single crystals at the nanoscale by varying the relative humidity level in a scanning probe microscopy chamber. The solution of the corresponding coupled electro-mechanical boundary problem allows explaining observed competition between ferroelastic and ferroelectric domain growth. The reduction in the ferroelastic domain density during local switching at elevated humidity has been attributed to changes in the electric field spatial distribution and screening effectiveness. The established mechanism is important because it reveals a kinetic nature of the final domain patterns in multiaxial materials and thus provides a general pathway to create desirable domain structure in ferroelectric materials for applications in piezoelectric and optical devices.

## 1. Introduction

Multiaxial ferroelectrics exhibit a coupling between spontaneous polarization and stress, which results in the formation of the ferroelastic and ferroelectric domain walls. The ferroelectric domain walls separate domains with opposite polarization directions, while domain walls with another angle between polarization directions are ferroelastic. The ferroelectric domain wall motion is activated by the application of the external electric field and is generally insensitive to mechanical stress, while ferroelastic domain walls react on both electric and elastic fields [1]. The ability of domain walls to be created or erased under external stimuli allows to consider them as tunable elements in the nanoelectronic devices. The electric, electromechanical, and thermal properties of ferroelectrics can be precisely controlled by assembling the domain patterns consisted of walls with varying densities, types, and charge states [2,3,4,5,6,7,8,9,10]. As an example, the periodical domain structure with 180-degree walls is used for the production of the efficient nonlinear-optical frequency converters [11], whereas the ferroelastic switching mediates extra-large piezoelectric response [12,13].

The dynamics of the 180-degree domain walls is comprehensively studied in the uniaxial crystals [14,15]. The polarization reversal, in this case, can be considered as a first-order phase transition between two states with opposite directions of spontaneous polarization, and the polar component of the electric field is the driving force of the process. Domain structure evolution during polarization reversal proceeds through the following stages: (1) domain nucleation, (2) forward domain growth in polar direction, (3) sideways domain wall motion, and (4) domain coalescence [11]. Despite the existence of strong background in studies of the barium titanate classical multiaxial ferroelectric [16] and apparent similarity of the ferroelastic and ferroelectric switching process, far less is known about ferroelastic switching [17,18]. According to the macroscopic experiments, the dynamics of ferroelectric and ferroelastic domain walls alternate during switching, and these processes have different characteristic times [19,20]. In piezoelectric ceramics, the domination of ferroelectric or ferroelastic switching events in the total switching, i.e., 180-degree and non-180-degree polarization reversal, is still under discussion yet [20,21]. In situ optical and electron microscopy visualizations conclusively approved that 180-degree polarization reversal in the single crystals and thin films of ferroelectrics from *c*^+^-domain with upward-directed polarization to the *c*^–^-domain with downward-directed polarization predominantly occurs through the intermediate state of ferroelastic domain wall formation (growth of the *a-*domain), i.e., two 90-degree switching events in [001] cut tetragonal ferroelectrics [21,22,23,24], or subsequent 71°, 109°, and 180° switching events in [111] cut rhombohedral ferroelectrics [25,26,27]. Landau–Ginzburg–Devonshire calculations explain such behavior as an existence of the local energetical minimum between upward and downward-directed polarization states, associated with the formation of *a-*domains [28,29,30]. The transition between two global energetical minima usually occurs through the local minima due to lower energetical barriers [28]. The direct 180-degree polarization reversal is, by all appearances, possible in the case where the formation of the ferroelastic domains is hindered because of some reason, such as for strong film clamping to the substrate mediated by the compressive stress or in the local switching by the biased tip of the scanning probe microscope (SPM), where the switching volume is clamped by the unswitched matrix [31,32,33,34]. On the other hand, ferroelastic domain patterns are usually metastable and relax to the more energetically favorable *c*^+^- or *c*^–^-domain states on some timescale [29]. The primary source of this relaxation comes from the mechanical stresses accumulated as a result of *a*-domain formation [23,35], while their release can significantly slow down the relaxation process [29,36].

Regardless of the discussed energy-conditioned driving forces of the ferroelectric–ferroelastic switching, the polarization reversal process is significantly determined by kinetic factors: screening, the interaction between domain walls, etc. [30]. For example, the inhomogeneous electric field created by the biased tip can significantly change the ferroelectric and ferroelastic switching dynamics and create energetically non-favorable patterns of vortex-like domains [30,37]. Despite the different time constants, which is caused by the different threshold voltage for the *a-* and *c*-domain switching due to the significantly different energy barrier height [28,38], both processes can generally occur simultaneously, because of the local inhomogeneity of the electric field and mechanical stress [39]. Thereby, an interaction between *a-*domains [18], and between *a-* and *c-*domains [40], can occur, as well as some collective regimes of the domain dynamics [1,41,42,43].

In this work, we reveal the role of domain dynamics in the formation of the domain structure during local polarization reversal in the multiaxial ferroelectric crystals. By the example of (111) 0.72Pb(Mg_1/3_Nb_2/3_)O_3_-0.28PbTiO_3_ (0.72PMN-0.28PT) ferroelectric single crystals, we detangle the complicated dynamics of the ferroelectric and ferroelastic domain walls during the local switching by SPM probe. The competition between ferroelectric and ferroelastic switching mechanisms was explored, which is controlled by the value and distribution of the electric field from the SPM probe. In a dry atmosphere mediating high localization in the electric field, complicated domain patterns are formed, with both ferroelectric and ferroelastic domain walls. Increasing the relative humidity leads to a stronger and more homogeneous electric field, which realizes switching with the formation of an isolated ferroelectric domain with 180-degree domain walls. The finite element simulations of the spatial distribution of the electric field and accompanying electromechanical stress allowed the electric field components to be studied, which drives local polarization reversal at the different stages of the domain dynamics. It was demonstrated that the formation of the 180-degree domain walls significantly reduces the stress components, thereby impeding ferroelastic switching. The competition between purely ferroelectric and ferroelectric–ferroelastic switching was revealed, which illustrates different possible pathways of polarization reversal in the multiaxial ferroelectrics controlled by switching kinetics.

## 2. Experimental Section

### 2.1. Sample Synthesis and Preparation

The studied 72PMN-28PT single crystals were grown in Xi’an Jiaotong University, China, using the modified Bridgman technique. The details of sintering can be found elsewhere [26]. Crystalline plates, with thicknesses of 0.5 mm and 1 mm, were cut perpendicular to [111] from the boule. The samples were identified to be of rhombohedral *3m* symmetry at room temperature. The single-domain state was achieved using a rectangular field pulse with an amplitude of 300 V/mm of 10 s duration at room temperature (we further refer this orientation of the polarization as *c*↑, according to Table 1). The samples were glued to the metal disks with a conductive silver paste.

### 2.2. Piezoresponse Force Microscopy and Local Switching

Domain imaging on the surface with the high spatial resolution was performed by piezoresponse force microscopy (PFM) using a scanning probe microscope NTEGRA Aura (NT-MDT, Russia) and MFP-3D-SA (Oxford Instruments, UK). The silicon NSC18 tips (MikroMash, Bulgaria) with Ti/Pt conductive coating and a 35 nm nominal radius of curvature were used. 1.5 V AC voltage at a frequency near the first flexural contact resonance was applied to the tip. The variable relative humidity was controlled using a self-made humidifier.

The macro-scale switching was carried out using a tungsten probe with the etch-sharpened tip (radius around 10 μm) as the top electrode and the saturated aqueous solution of lithium chloride as the bottom one. The triangular voltage pulse with a 40 V/s rate and a 2 s duration was generated with PCI-6251 multifunction input-output device (National Instruments Corp., USA) and amplified by a high-voltage amplifier 677B (Advanced Energy Industries Inc., USA). The switching current was recorded using the self-made operational amplifier ammeter. The optical domain imaging was carried out using the optical microscope LMA10 (Carl Zeiss AG, Germany) and the high-speed Mini UX100 camera (Photron Ltd., Japan).

### 2.3. Finite Element Simulations

The spatial distribution of the polar component of the electric field (*E_z_*) and mechanical stress (*σ*) produced by a biased conductive SPM tip was calculated by the solution of the coupled electrical and mechanical equations in the approximation of the static problem. The simulations were performed using the finite element method in the commercial software COMSOL Multiphysics (“AC/DC electrostatics” and “Structural mechanics” modules). The relative permittivity, elastic, and piezoelectric coefficients for the (111) PMN-PT were taken from Ref. [44]. The considered SPM tip had a cone-shaped geometry with a semispherical end and a circular disk-type contact area. The height of the tip conical part was 3 μm, the cone angle was 40 degrees, the sphere radius was 30 nm, and the radius of the contact area was 15 nm. The positive constant bias voltage of 90 V was applied to the tip while the solid bottom electrode was grounded. The sample was modeled as a cylinder with a 200 μm radius and a 100 μm thickness. The influence of the water meniscus which appeared at high-humidity conditions was simulated similarly to that in Refs. [45,46]. The height of the elliptical shape water meniscus has been varied from 50 to 1000 nm. The electric field in the presence of the *c-*domain was modeled by introducing the cylindrical-shaped *c*↓-domain under the probe with a radius ranging from 50 to 1000 nm.

## 3. Results

### 3.1. Local Switching in the Dry Atmosphere 

**Figure 1:**
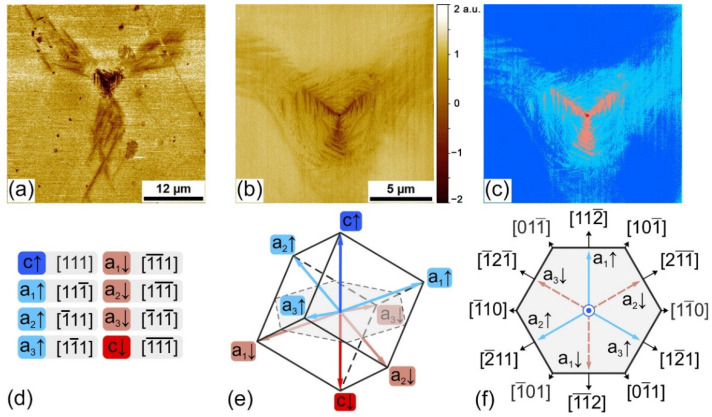
Local switching by 90 V, with a 500 ms voltage pulse in the dry atmosphere. The PFM images of different domain structures: (**a**) full-scale image and (**b**) central region. (**c**) Schematic illustrating orientation of the polarization of image (**b**). (**d**,**e**) Polarization notation and orientation used in the schematic (**c**). (**f**) Characteristic directions at (111) plane for an [111] oriented rhombohedral cell.

We started with local switching in the *c*↑*-*domain state in a dry atmosphere. The application of voltage pulses to the SPM tip resulted in the formation of the complicated pattern of *c-* and *a-*domains (Figure 1a–c). The domain structure (Figure 1b,d,f) consisted of: (1) the inner *c↓* domain with a radius of about 500 nm; (2) the web-like structure of *a↓*-domains oriented along $\left[\bar{11}2\right]$, $\left[\bar{1}2\bar{1}\right]$, and $\left[2\bar{11}\right]$ crystallographic directions, covering the area with a radius of about several microns; and (3) the “leaf-like” areas with weak PFM contrast that expands in the same crystallographic directions at the distance up to 20 μm from the point of voltage application. The analysis of the shape and orientation of the domains in the PFM images allows four domain types to be separated: the initial *c*↑ domain, and the reversed *c*↓-, *a*↑-, and *a*↓-domains (Figure 1e,f). The whole domain pattern grew with the increase of the voltage pulse amplitude and duration (see Supplementary Materials, Figure S2). The observed behavior drastically differs from that of earlier performed local switching experiments in the multiaxial ferroelectric crystals [47,48,49]. The evident change of the surface topography in the *a*-domain area has been revealed, which is due to a rise in mechanical stress (Supplementary Materials, Figure S3).

**Figure 2:**
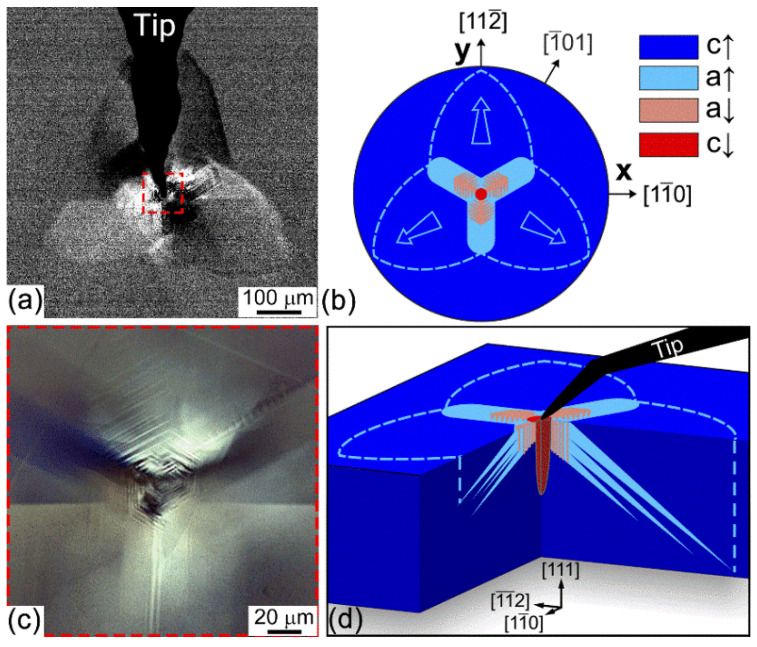
(**a**) Optical image of the domain structure obtained during the application of 96 V to the probe. (**b**) Schematic representation of the obtained domain structure at the surface. (**c**) Static optical image of the zoom region near the tip from structure showed at (**a**) 24 h after the experiment. (**d**) Schematic volumetric reconstruction of the obtained domain structure.

To obtain insight into the domain structure evolution, the local switching experiments were reproduced with a 10 µm radius probe, and the domain patterns were visualized using polarized optical microscope (Figure 2a,c). Much larger sizes of the probe, as compared with the SPM tip, allow large domain patterns to be created, which can be visualized with a high enough spatial resolution at the surface and in the bulk of the crystal. The domain structure evolution in these experiments represents the growth of the leaf-like areas spreading in the $\left[11\bar{2}\right]$, $\left[1\bar{2}1\right]$, and $\left[\bar{2}11\right]$ directions. Optical imaging of the domain structure by the objective with higher magnification (×100) reveals similar domain patterns as by PFM (Figure 2c). By considering the possible directions of the *a-*domain growth (Figure 2b,d), weak PFM contrast can be attributed to the intersections of *a*↑-domains and the sample surface. The optical contrast of leaf-like areas is an overlay of the optical contrasts from the individual wide *a*↑-domain wedges inclined to the bulk at a 35.5° angle (Figure 2d) [25].

The revealed kinetics of the polarization reversal are similar to those obtained earlier for switching by the uniform electric field in the same crystals, where switching from the *c*↑- to *c*↓-domain state was realized through two successive stages: *a*↑- and *a*↓- domain states [25], resulting from the lower potential barrier of the *a-*domain formation [28]. Nonetheless, the observed complex domain patterns revealed by PFM cannot be simply interpreted as a transitional state between *c*↑- and *c*↓-domain states because the increase in the voltage pulse duration and amplitude does not result in the “complete” reversal to the *c*↓-domain state (see Supplementary Materials, Figure S1).

**Figure 3:**
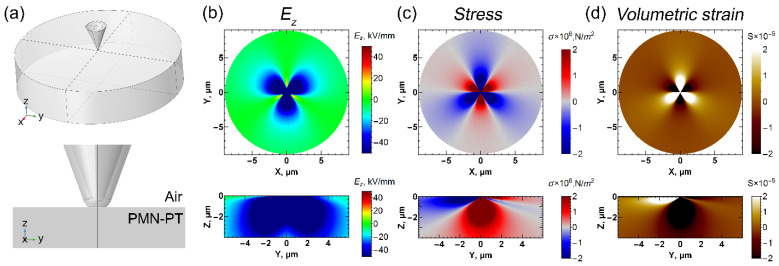
COMSOL modeling for the dry atmosphere: (**a**) scheme, (**b**) logarithm of the electric field polar component, (**c**) stress, (**d**) volumetric strain.

In order to understand the domain structure evolution during polarization reversal, the distributions of electric field and mechanical stress in the vicinity of the SPM tip were analyzed using finite element modeling (FEM) simulations of the respective boundary problem (Figure 3a). The simulations show a highly anisotropic electric field and mechanical stress distribution in rhombohedral PMN-PT, associated with the electro-elastic coupling and piezo-polarization (Figure 3b,c). As the first transition from the *c*↑*-*domain state to the *a*↑*-*domain state is caused by the polarization decrease in the (111) direction of the lattice, it is reasonable to conclude that the *E_z_* component of the electric field should start the switching process. The field maximum is localized near the tip contact point. The corresponding distributions of the stress and volumetric strain are presented in Figure 3c,d. In the direction of the maximum electric field *E_z_* component at (111) plane (which is the set of $\left[\bar{11}2\right]$, $\left[\bar{1}2\bar{1}\right]$, and $\left[2\bar{11}\right]$ crystallographic directions), the mechanical stress is positive (Figure 3c, red color), which corresponds to the surface compression (Figure 3d, dark contrast), while in the directions rotated at a 60° angle (set of $\left[11\bar{2}\right]$, $\left[1\bar{2}1\right]$, and $\left[\bar{2}11\right]$ directions), the field is close to zero, and mechanical stress is negative (Figure 3c, blue color), i.e., it corresponds to the expansion at the surface (Figure 3d, bright contrast). 

A comparison between the *E_z_* component of the electric field and mechanical stress distribution (Figure 3b,c), and the domain pattern formed after the local switching (Figure 1a,b) shows that the structure of *a*-domains is localized in the area with positive mechanical stress, while the switching does not occur in the negatively stressed areas. The *c↓-*domain only forms in the vicinity of the point of voltage pulse application, where a maximum of the electric field is localized. Thus, *c↓-*domain formation occurs in the highest electric field, while *a-*domains appear in the much larger area, which can be explained by the significantly lower threshold voltages.

### 3.2. Local Switching in Humid Conditions

**Figure 4:**
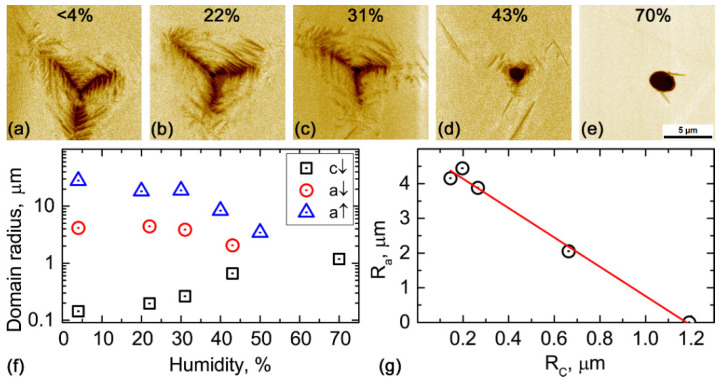
Local switching at different relative humidity: (**a**) < 4%, (**b**) 20%, (**c**) 30%, (**d**) 40%, and (**e**) 70%. (**f**) Dependence of domain size on relative humidity. (**g**) Dependence of the mean radius of *a*↓-domains on the radius of the central *c*-domain. Voltage 90 V, pulse duration 500 ms.

As was shown in the previous paragraph, *c↓* domains were formed in a much stronger electric field near the point of the electric field application. To control the field distribution under the SPM tip, the relative humidity (RH) can be tuned as it is known to affect the distribution of the electric field from the SPM tip and maximize *E_z_* in the area near the contact [45]. Increasing the humidity surprisingly eliminates *a-*domains, and pure ferroelectric switching is realized for RH > 40% (Figure 4).

**Figure 5:**
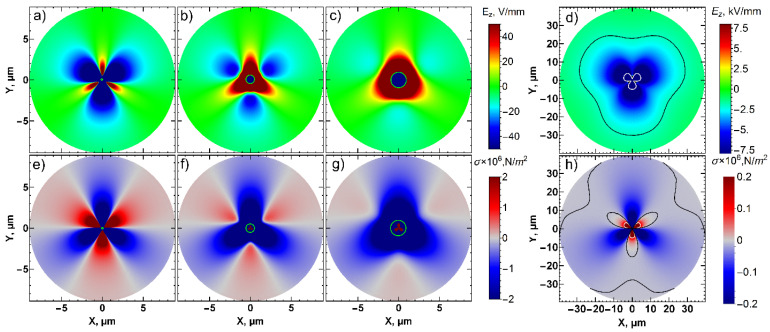
Simulated distributions of (**a**–**d**) E_z,_ and (**e**–**h**) mechanical stress, s, at the 20 nm depth with different radius of the *c*-domain in the center (green circle): (**a**,**d**) 50 nm, (**b**,**e**) 500 nm, (**c**,**f**) 1000 nm. (**d**,**e**) 50 nm, larger scale. The green line corresponds to the *c*-domain wall. The black line corresponds to threshold field for the *a*↑-domain ~ −2 V/mm (1.4 × 10^5^ N/m^2^ stress), while the white line corresponds to the threshold field for *a*↓-domains ~ −20 V/mm (7 × 10^3^ N/m^2^ stress).

Notably, the introduction of the water meniscus at the tip-sample interface in the FEM leads to an increase in the absolute values of the electric field and the stress under the probe, but anisotropic distribution remains the same as in the dry atmosphere (Supplementary, Figure S4). To explain the observed switching behavior, the small-size *c-*domain was added under the SPM tip in FEM (Figure 5a–c,e–g, green circle). In this case, the values of the positive stress and negative *E_z_* significantly decrease. When the radius of the introduced *c-*domain exceeds the radius of the meniscus, the positive stress component decreases to negligible values. Thus, the appearance of the *c-*domain under the tip leads to the compensation of the elastic field with the release of mechanical stress, responsible for the formation of *a*-domains. The corresponding distribution of *E_z_* and mechanical stress, *σ*, in the bulk of the crystal can be found in Figures S4–S6 of the Supplementary Information.

## 4. Discussion

Polarization reversal by the uniform electric field always starts with the formation of *a-*domains, while *c-*domains with opposite orientation appear under a much stronger electric field [25]. Based on the presented experimental data and results of the modeling, similar behavior can be traced in most of the switching areas, except the immediate position of the SPM probe. As an electric field is notably high in the vicinity of the SPM tip (up to 10^9^ V/m according to simulations), the formation of the *c↓*-domain is likely in this area, but a further lateral expansion of the created domain is limited by the radius of the tip/meniscus where the maximum electric field is concentrated. *a*-domains can propagate at longer distances from the probe due to the much lower threshold field of such polarization reversal. As the SPM tip creates a non-uniform electric field, the threshold voltage for *a-*domain formation can be evaluated by comparing the average distance of the domain propagation and local values of the electric field at this given distance from the tip. The values of the surface area occupied by the *c*↓-, *a*↑-, and *a*↓-domains for the different humidity conditions were extracted from the experimental dependencies as a maximal distance from the SPM probe (averaged by the three branches), where respective domain structure was formed (Figure S7 and Figure 4f). The radii of the areas with *a*-domains were used to calculate the threshold field of the polarization reversal for *a*↑- and *a*↓-domains. The threshold field values were plotted over the distribution of the electric field and stress in Figure 5d,h (black and white lines, respectively). The threshold field for *a*↑-domains was found to be around 2 V/mm, while for *a*↓-domains, it achieves –20 V/mm. Note, however, that the last value represents only a rough estimation as the distribution changes significantly after the formation of *a*↑-domains. As the threshold voltage for polarization reversal is assumed to be significantly determined by the energy barriers for the rearrangement of the crystalline lattice, the energy barriers to “compress” and “rotate” the polarization are thought to be different [28]. It should be noted that obtained threshold electric field values for the formation of *a-*domains are almost an order lower than the threshold field for the *c-*domain formation determined from the macroscopic experiment [25]. 

**Figure 6:**
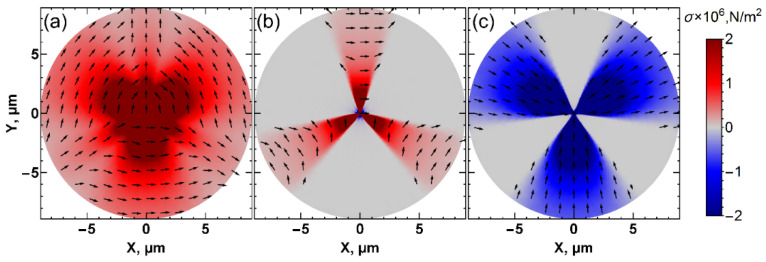
The distributions of (**a**) σ_1_, (**b**) σ_2_, and (**c**) σ_3_ principal components of the stress tensor at the 20 nm depth. The black arrows indicate strain direction.

The total stress values at the respective distances for *a*↑- and *a*↓-domains are 7·kPa and 140 kPa, respectively. A clearer understanding of the contribution of stress to ferroelastic switching can be extracted from the principal components of stress (Figure 6). The arrows in Figure 6 depict the direction of the deformation vector (compression/expansion), while the color indicates that red (positive) is compression and blue (negative) is expansion. It is seen that *σ*_1_, the main component, is responsible for the switching. The lattice contraction in the directions perpendicular to the three main branches of the domain pattern in Figure 1a results in the formation of the domain with orthogonally oriented polarization. The principal stress *σ*_1_ behaves differently in three other symmetry directions, where the formation of *a*↑-domains was not observed (Figure 6a). The contraction of the lattice goes along with these directions, which impedes the formation of ferroelastic domains. The second principal component of the stress tensor, *σ_2_*, has contributed to the crystal directions, where the switching is not observed. The maximum of the third principal component, σ_3_, also corresponds to the symmetry of the switched domain patterns, which indicates an expansion of the lattice in these three directions under the action of the orthogonal compression. An evaluation of the threshold stress values for the *σ_1_* principal component gives 100 kPa for *a*↑-domains and 1750 kPa for *a*↓-domains, respectively. The contribution to the switching from the different components of the stress tensor is not so apparent (Figure S7). However, it can be concluded that in-plane components (11, 12, 21, and 22) are significantly larger than out-of-plane (33, 32, 23, 31, and 13) components, which indicates their larger contribution to switching.

The switching kinetics should be discussed in more detail. At the first stage, the transition from *c*↑- to *a*↑-domain states occurs in a weak electric field. This type of switching is caused by the necessity to compensate mechanical energy which appears as a result of the crystal contraction in the electric field. This process is truly ferroelastic switching and it differs from the normal switching in the uniaxial ferroelectric material, because the transition occurs without polarization rotation but with the lattice contraction and a decrease in the polarization magnitude. The difference can also be illustrated by a comparison of the normal component and electric field components along $\left[11\bar{2}\right]$, $\left[1\bar{21}\right]$, and $\left[\bar{2}11\right]$ crystallographic directions of the crystal (along the polarization direction in *a*↑-domains, see Supplementary Materials Figure S8). We further term this direction as the “71°-degree direction of the electric field”. It is seen that the anisotropic growth of *a-*domains specifically occurs in the directions determined by the normal component of the electric field, while the 71° component of the positive sign, necessary for the polarization reversal, is stronger in the directions where domains are not formed. As such, the kinetics of the polarization reversal is likely to occur under the *E_z_* component, but not by the 71° component of the electric field.

On the other hand, the formed *a-*domains have a lower polarization projection value with respect to the crystal surface, which is connected to the larger change in the polarization magnitude, i.e., the amount of necessary screening charge. An increase in humidity enlarges the meniscus area and the electric field in the region around the tip above the threshold voltages for *a↓-* and *c*-domains, which stimulates the growth of *c* domains underneath the SPM tip. The appearance of the *c↓-*domains naturally limits the formation of *a*-domains by the partial release of mechanical stress. The formation and growth of the *c↓-* and *a*-domains are realized simultaneously, and the final domain pattern is a result of the self-consistent change in the distribution of electric and elastic fields during the *c*-domain growth. The formation of *a-*domain partly compensates stress caused by piezoelectric polarization. At the same time, *c* domains form under the much stronger electric field, but their formation also compensates the mechanical stress and thereby further impedes the propagation of *a-*domains. Kinetic competition between the growth of the *a*- and *c-*domains is responsible for the formation of the experimentally observed complicated domain patterns. It should be noted that the competition is not only determined by the distribution of the electric field and mechanical stress, but also by screening conditions. The propagation of the screening charge at mm-scale distances is complicated and needs longer times. Thus, *c*-domain appears in the vicinity of the SPM tip/meniscus when the value of the electric field exceeds the threshold voltage for the polarization reversal and screening conditions are granted.

The discussed mechanism can be applied for the macroscopic polarization reversal in the multiaxial materials without the loss of generality. The 180-degree and non-180-degree domain wall motion processes are demonstrated to compete during the polarization reversal. Therefore, polarization reversal should be significantly dependent on the local conditions, such as the local mechanical stresses and nonuniform distribution of the electric field. The measurements were performed in the crystals with 0.72PMN-0.28PT composition because they possess a large electromechanical coupling coefficient, and the composition is still away from the morphotropic phase boundary at room temperature [50]. This case is the most apparent because it allows performing calculations for the pure rhombohedral phase. Also, switching through the *a*-domain formation is easier to be realized in a crystal with a large electromechanical coupling coefficient. Nevertheless, according to the theoretical predictions, the researched effects are general, and the results should be valid for other ferroelectric–ferroelastic materials. The pathway of the polarization reversal, threshold fields, degree of anisotropy, switched areas, etc. should be different. It is expected that larger-scale ferroelastic switching is possible for materials with larger electromechanical coupling. Revealing further dependencies of the switching parameters on the properties of the crystals is a matter of further study.

## 5. Conclusions

In this paper, we observed the competitive switching dynamics of the ferroelectric and ferroelastic domain walls in the rhombohedral (111) cut PMN-PT single crystals. The circular-shaped ferroelectric domain formed immediately underneath the tip, while irregular-shaped ferroelastic domain patterns widely spread tens of microns far from the tip position. The possibility of controlling the switching process using varied relative humidity (RH) levels was demonstrated. For RH > 40%, the switching leads to the formation of the *c-*domains only, while for RH < 40%, the formation of a mixture of *a*- and *c*-domain states was observed. The difference of the kinetics in these experiments can be explained by the variation of the threshold voltages for the *c*- and *a*-domains nucleation and the influence of the water meniscus underneath SPM probe on the distribution of the electric field. The *c-*domains appear in the highest electric field in the vicinity of the tip/meniscus. Formation of the *c-*domains impedes further *a-*domain switching because of the mechanical stress release. Thus, the ferroelectric–ferroelastic switching behavior is significantly dependent on the distribution of the electric field, mechanical stress, and screening conditions.

## Figures and Tables

**Table 1:** Notations for the spontaneous polarization vector for the [111] oriented rhombohedral cell.

Vector of Spontaneous Polarization	Notation	Vector of Spontaneous Polarization	Notation
[111]	*c**↑***	$\left[\bar{111}\right]$	*c**↓***
$\left[11\bar{1}\right]$	*a*_1_ ***↑***	$\left[\bar{1}\bar{1}1\right]$	*a*_1_ ***↓***
$\left[\bar{1}11\right]$	*a*_2_ ***↑***	$\left[1\bar{1}\bar{1}\right]$	*a*_2_ ***↓***
$\left[1\bar{1}1\right]$	*a*_3_ ***↑***	$\left[\bar{1}1\bar{1}\right]$	*a*_3_ ***↓***

## Data Availability

Data are contained within the article or supplementary material.

## Supplementary Materials

The following supporting information can be downloaded at: https://www.mdpi.com/article/10.3390/nano12213912/s1. Figure S1: Optical images of the domain structure evolution under the tip under rising voltage. Figure S2: Results of the local switching with different applied amplitudes and voltage pulse durations. Figure S3: PFM images of topography changes after local switching and of the formed domain pattern. Figure S4: Simulated electric field and strain distributions at a 20 nm depth with different meniscus heights. Figure S5: Simulated distributions of the vertical component of the electric field at the 20 nm depth with different radii of the *c* domain in the center. Figure S6: Simulated distributions of mechanical stress at the 20 nm depth with different radii of the *c* domain in the center. Figure S7: Schematics, illustrating, how the effective radiuses of the domain were derived in Figure 4f, in the main body of the paper. Figure S8: The distributions of stress tensor components at the 20 nm depth. Figure S9: Simulation of the 71-degree components of the electric field (along the polarization direction in *a*↑ domains).

## References

[1] Scott J.F., Hershkovitz A., Ivry Y., Lu H., Gruverman A., Gregg J.M. (2017). Superdomain dynamics in ferroelectric-ferroelastic films: Switching, jamming, and relaxation. Appl. Phys. Rev..

[2] Seidel J., Martin L.W., He Q., Zhan Q., Chu Y.-H., Rother A., Hawkridge M.E., Maksymovych P., Yu P., Gajek M. (2009). Conduction at domain walls in oxide multiferroics. Nat. Mater..

[3] Burns S.R., Tselev A., Ievlev A.V., Agar J.C., Martin L.W., Kalinin S.V., Sando D., Maksymovych P. (2022). Tunable microwave conductance of nanodomains in ferroelectric PbZr_0.2_Ti_0.8_O_3_ thin film. Adv. Electron. Mater..

[4] McConville J.P.V., Lu H., Wang B., Tan Y., Cochard C., Conroy M., Moore K., Harvey A., Bangert U., Chen L.-Q. (2020). Ferroelectric domain wall memristor. Adv. Funct. Mater..

[5] Lu H., Tan Y., McConville J.P.V., Ahmadi Z., Wang B., Conroy M., Moore K., Bangert U., Shield J.E., Chen L.-Q. (2019). Electrical tunability of domain wall conductivity in LiNbO_3_ Thin Films. Adv. Mater..

[6] Rojac T., Ursic H., Bencan A., Malic B., Damjanovic D. (2015). Mobile domain walls as a bridge between nanoscale conductivity and macroscopic electromechanical response. Adv. Funct. Mater..

[7] Hopkins P.E., Adamo C., Ye L., Huey B.D., Lee S.R., Schlom D.G., Ihlefeld J.F. (2013). Effects of coherent ferroelastic domain walls on the thermal conductivity and Kapitza conductance in bismuth ferrite. Appl. Phys. Lett..

[8] Liu C., Chen Y., Dames C. (2019). Electric-field-controlled thermal switch in ferroelectric materials using first-principles calculations and domain-wall engineering. Phys. Rev. Appl..

[9] Qiu C., Wang B., Zhang N., Zhang S., Liu J., Walker D., Wang Y., Tian H., Shrout T.R., Xu Z. (2020). Transparent ferroelectric crystals with ultrahigh piezoelectricity. Nature.

[10] Bokov A.A., Ye Z.-G. (2006). Recent progress in relaxor ferroelectrics with perovskite structure. J. Mater. Sci..

[11] Shur V.Y., Akhmatkhanov A.R., Baturin I.S. (2015). Micro- and nano-domain engineering in lithium niobate. Appl. Phys. Rev..

[12] Hu Y., You L., Xu B., Li T., Morris S.A., Li Y., Zhang Y., Wang X., Lee P.S., Fan H.J. (2021). Ferroelastic-switching-driven large shear strain and piezoelectricity in a hybrid ferroelectric. Nat. Mater..

[13] Huang F., Hu C., Zhou Z., Meng X., Tan P., Wang Y., Wang C., Tian H. (2020). Improving strain in single crystal by composition-gradients design. Acta Mater..

[14] Lang S.B., Chan H.L.W. (2007). Frontiers of Ferroelectricity.

[15] Tagantsev A.K., Fousek F., Cross L.E. (2010). Domains in Ferroic Crystals and Thin Films.

[16] Jona F., Shirane G. (1993). Ferroelectric Crystals.

[17] Salje E.K.H. (2012). Ferroelastic Materials. Annu. Rev. Mater. Res..

[18] Ignatans R., Damjanovic D., Tileli V. (2021). Individual Barkhausen Pulses of Ferroelastic Nanodomains. Phys. Rev. Lett..

[19] Daniels J.E., Cozzan C., Ukritnukun S., Tutuncu G., Andrieux J., Glaum J., Dosch C., Jo W., Jones J.L. (2014). Two-step polarization reversal in biased ferroelectrics. J. Appl. Phys..

[20] Schultheiß J., Liu L., Kungl H., Weber M., Kodumudi Venkataraman L., Checchia S., Damjanovic D., Daniels J.E., Koruza J. (2018). Revealing the sequence of switching mechanisms in polycrystalline ferroelectric/ferroelastic materials. Acta Mater..

[21] Jiang B., Bai Y., Chu W., Su Y., Qiao L. (2008). Direct observation of two 90° steps of 180° domain switching in under an antiparallel electric field Direct observation of two 90° steps of 180° domain switching in BaTiO_3_ single crystal under an antiparallel electric field. Appl. Phys. Lett..

[22] Khan A.I., Marti X., Serrao C., Ramesh R., Salahuddin S. (2015). Voltage-Controlled ferroelastic switching in Pb(Zr_0.2_Ti_0.8_)O_3_ thin films. Nano Lett..

[23] Xu R., Liu S., Grinberg I., Karthik J., Damodaran A.R., Rappe A.M., Martin L.W. (2015). Ferroelectric polarization reversal via successive ferroelastic transitions. Nat. Mater..

[24] Damodaran A.R., Pandya S., Agar J.C., Cao Y., Vasudevan R.K., Xu R., Saremi S., Li Q., Kim J., McCarter M.R. (2017). Three-state ferroelastic switching and large electromechanical responses in PbTiO3 thin films. 2017, 29, 1702069. Adv. Mater..

[25] Ushakov A.D., Esin A.A., Akhmatkhanov A.R., Hu Q., Liu X., Zhao Y., Andreev A.A., Wei X., Shur V.Y. (2019). Direct observation of domain kinetics in rhombohedral PMN-28PT single crystals during polarization reversal. Appl. Phys. Lett..

[26] Ushakov A.D., Turygin A.P., Akhmatkhanov A.R., Alikin D.O., Hu Q., Liu X., Zhao Y., Xu Z., Wei X., Shur V.Y. (2020). Dense ferroelectric-ferroelastic domain structures in rhombohedral PMN-28PT single crystals. Appl. Phys. Lett..

[27] Li M., Wang B., Liu H.-J., Huang Y.-L., Zhang J., Ma X., Liu K., Yu D., Chu Y.-H., Chen L.-Q. (2019). Direct observation of weakened interface clamping effect enabled ferroelastic domain switching. Acta Mater..

[28] Genenko Y.A., Khachaturyan R., Vorotiahin I.S., Schultheiß J., Daniels J.E., Grünebohm A., Koruza J. (2020). Multistep stochastic mechanism of polarization reversal in rhombohedral ferroelectrics. Phys. Rev. B.

[29] Baek S.H., Jang H.W., Folkman C.M., Li Y.L., Winchester B., Zhang J.X., He Q., Chu Y.H., Nelson C.T., Rzchowski M.S. (2010). Ferroelastic switching for nanoscale non-volatile magnetoelectric devices. Nat. Mater..

[30] Balke N., Choudhury S., Jesse S., Huijben M., Chu Y.H., Baddorf A.P., Chen L.Q., Ramesh R., Kalinin S.V. (2009). Deterministic control of ferroelastic switching in multiferroic materials. Nat. Nanotechnol..

[31] Rodriguez B.J., Eng L.M., Gruverman A. (2010). Web-like domain structure formation in barium titanate single crystals. Appl. Phys. Lett..

[32] Zhao K.Y., Zhao W., Zeng H.R., Yu H.Z., Ruan W., Xu K.Q., Li G.R. (2015). Tip-bias-induced domain evolution in PMN–PT transparent ceramics via piezoresponse force microscopy. Appl. Surf. Sci..

[33] Kholkin A.L., Bdikin I.K., Shvartsman V.V., Pertsev N.A. (2007). Anomalous polarization inversion in ferroelectrics via scanning force microscopy. Nanotechnology.

[34] Zhao B., Chen Z., Meng J., Lu H., Zhang D.W., Jiang A. (2015). Ferroelectric polarization and defect-dipole switching in an epitaxial (111) BiFeO_3_ thin film. J. Appl. Phys..

[35] Su D., Meng Q., Vaz C.A.F., Han M.-G., Segal Y., Walker F.J., Sawicki M., Broadbridge C., Ahn C.H. (2011). Origin of 90° domain wall pinning in Pb(Zr_0.2_Ti_0.8_)O_3_ heteroepitaxial thin films. Appl. Phys. Lett..

[36] Nagarajan V., Roytburd A., Stanishevsky A., Prasertchoung S., Zhao T., Chen L., Melngailis J., Auciello O., Ramesh R. (2003). Dynamics of ferroelastic domains in ferroelectric thin films. Nat. Mater..

[37] Béa H., Ziegler B., Bibes M., Barthélémy A., Paruch P. (2011). Nanoscale polarization switching mechanisms in multiferroic BiFeO 3 thin films. J. Phys. Condens. Matter.

[38] Baek S.H., Eom C.B. (2012). Reliable polarization switching of BiFeO_3_. Philos. Trans. R. Soc. A.

[39] Britson J., Nelson C., Pan X., Chen L.-Q. (2014). First-order morphological transition of ferroelastic domains in ferroelectric thin films. Acta Mater..

[40] Li W., Alexe M. (2007). Investigation on switching kinetics in epitaxial Pb(Zr_0.2_Ti_0.8_)O_3_ ferroelectric thin films: Role of the 90° domain walls. Appl. Phys. Lett..

[41] Ivry Y., Scott J.F., Salje E.K.H., Durkan C. (2012). Nucleation, growth, and control of ferroelectric-ferroelastic domains in thin polycrystalline films. Phys. Rev..

[42] Lu X., Chen Z., Cao Y., Tang Y., Xu R., Saremi S., Zhang Z., You L., Dong Y., Das S. (2019). Mechanical-force-induced non-local collective ferroelastic switching in epitaxial lead-titanate thin films. Nat. Commun..

[43] Sharma P., McQuaid R.G.P., McGilly L.J., Gregg J.M., Gruverman A. (2013). Nanoscale dynamics of superdomain boundaries in single-crystal BaTiO_3_ lamellae. Adv. Mater..

[44] Liu G., Jiang W., Zhu J., Cao W. (2011). Electromechanical properties and anisotropy of single- and multi-domain 0.72Pb(Mg_1/3_Nb_2/3_)O_3_-0.28PbTiO_3_ single crystals. Appl. Phys. Lett..

[45] Ievlev A.V., Morozovska A.N., Shur V.Y., Kalinin S.V. (2014). Humidity effects on tip-induced polarization switching in lithium niobate. Appl. Phys. Lett..

[46] Shishkina E.V., Pelegova E.V., Kosobokov M.S., Akhmatkhanov A.R., Yudin P.V., Dejneka A., Shur V.Y. (2021). Influence of humidity on local polarization reversal in a Rb:KTP single crystal. ACS Appl. Electron. Mater..

[47] Rodriguez B.J., Nemanich R.J., Kingon A., Gruverman A., Kalinin S.V., Terabe K., Liu X.Y., Kitamura K. (2005). Domain growth kinetics in lithium niobate single crystals studied by piezoresponse force microscopy. Appl. Phys. Lett..

[48] Ievlev A.V., Jesse S., Morozovska A.N., Strelcov E., Eliseev E.A., Pershin Y.V., Kumar A., Shur V.Y., Kalinin S.V. (2013). Intermittency, quasiperiodicity and chaos in probe-induced ferroelectric domain switching. Nat. Phys..

[49] Brugère A., Gidon S., Gautier B. (2011). Abnormal switching of ferroelectric domains created by the tip of an atomic force microscope in a congruent LiTaO_3_ single-crystal thin film. J. Appl. Phys..

[50] Liu G., Kong L., Hu Q., Zhang S. (2020). Diffused morphotropic phase boundary in relaxor-PbTiO_3_ crystals: High piezoelectricity with improved thermal stability. Appl. Phys. Rev..

