# Supplementary material for: Competition between Ferroelectric and Ferroelastic Domain Wall Dynamics during Local Switching in Rhombohedral PMN-PT Single Crystals
Source: Nanomaterials (Basel). 2022 Nov 6;12(21):3912. doi: 10.3390/nano12213912 (PMC9659027; doi:10.3390/nano12213912)
Supplement: Supplementary file 1 [file nanomaterials-12-03912-s001.zip › nanomaterials-1991872-supplementary.pdf]

Supplementary Materials

Competition between Ferroelectric and Ferroelastic Domain Wall Dynamics during Local Switching in Rhombohedral PMN-PT Single Crystals

Denis Alikin [1,*], Anton Turygin [1], Andrei Ushakov [1], Mikhail Kosobokov [1], Yurij Alikin [1], Qingyuan Hu [2], Xin Liu [2], Zhuo Xu [2], Xiaoyong Wei [2] and Vladimir Shur [1]

[1] School of Natural Sciences and Mathematics, Ural Federal University, 620000 Ekaterinburg, Russia
[2] Electronic Materials Research Laboratory, Key Laboratory of the Ministry of Education & International Center for Dielectric Research, Xi'an Jiaotong University, Xi'an 710049, China
* Correspondence: denis.alikin@urfu.ru

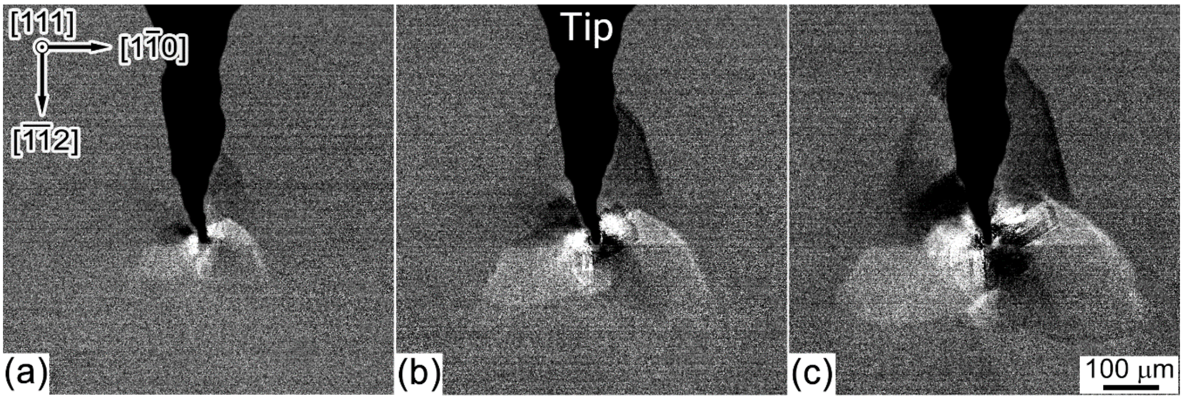

Figure S1. Optical images of the domain structure evolution under the tip under rising voltage (rate 40 V·s^{-1}): (a) 1 s; (b) 1.5 s; (c) 1.9 s since the pulse start.

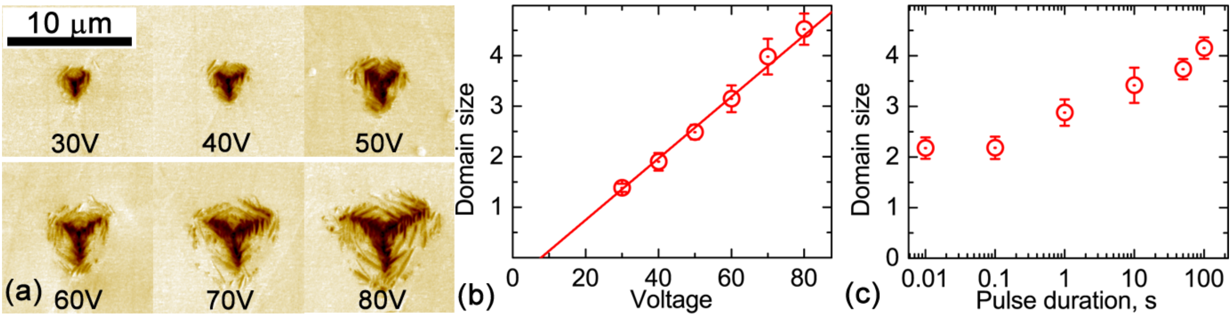

Figure S2. Results of the local switching with different applied amplitude and duration of the voltage pulses. (a) PFM images. Dependencies of a↓ domain size on (b) amplitude and (c) duration of the voltage pulses. Dry atmosphere.

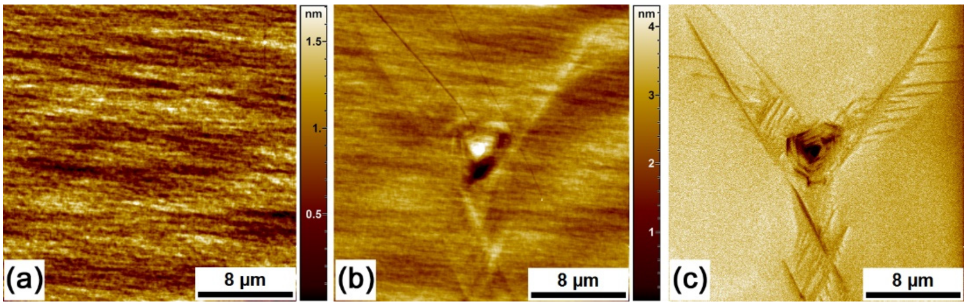

Figure S3. Topography changes after local switching. Topography (a) before and (b) after local switching. (c) PFM image of the domain pattern formed after 100V, 500 ms voltage pulse application. Dry atmosphere.

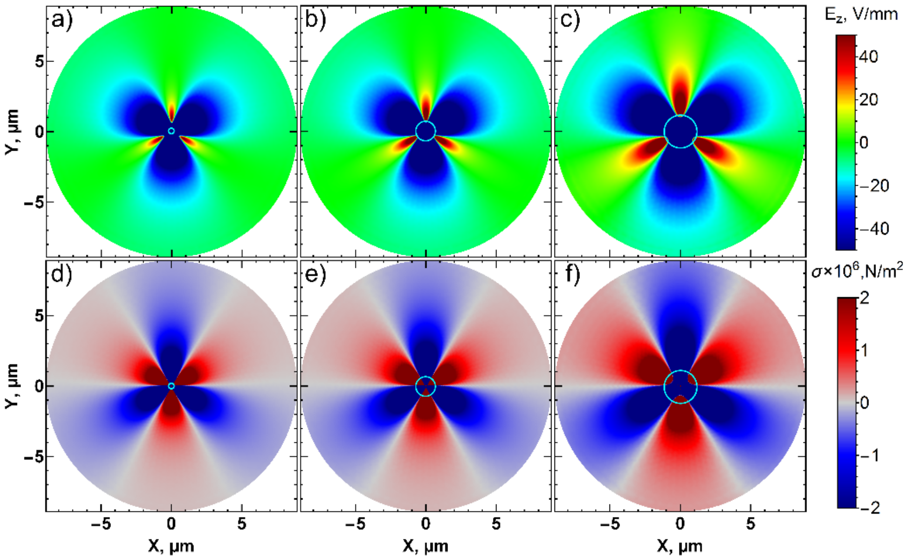

Figure S4. Simulated E_z (a-c) and σ (d-f) distributions at the 20 nm depth with different meniscus height (blue circle represents meniscus at the sample surface): a,d – h = 50 nm, b,e – h = 200 nm, c,f – h = 800 nm.

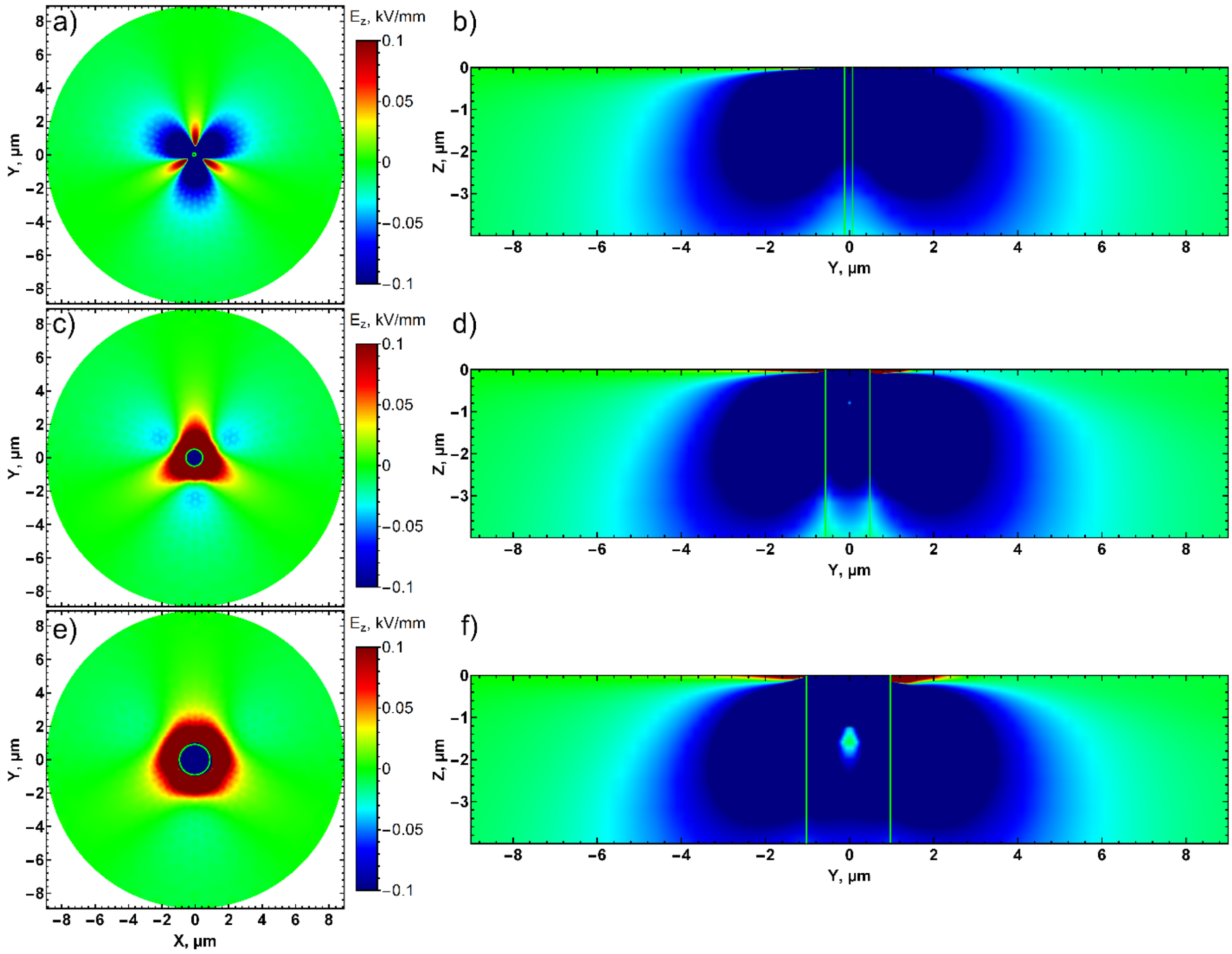

Figure S5. Simulated distributions of vertical component of the electric field, E_z, at the 20 nm depth with different radii of the c-domain in the center (green circle): (a,b) 50 nm, (c,d) 500 nm, (e,f) 1000 nm. Green lines correspond to the c-domain walls.

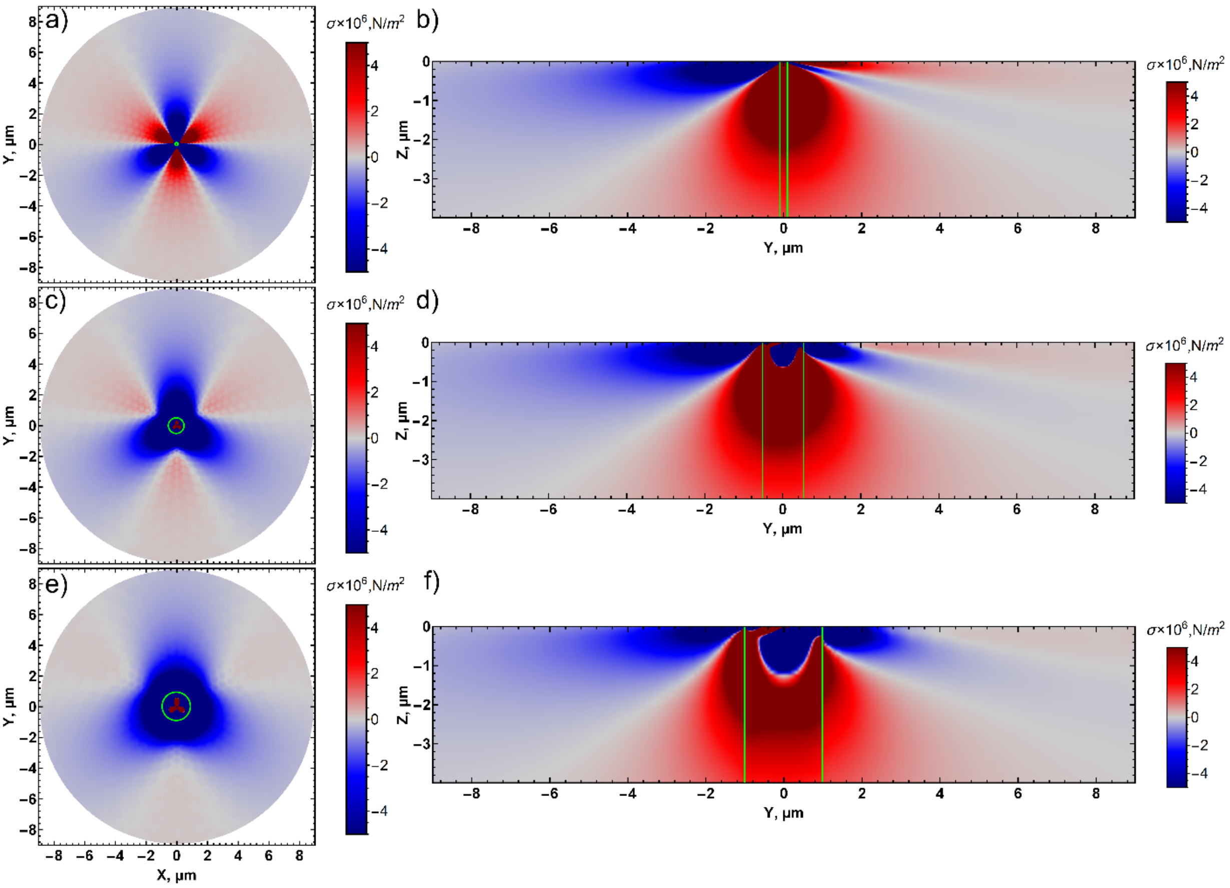

Figure S6. Simulated distributions of mechanical stress, σ, at the 20 nm depth with different radii of the *c*-domain in the center (green circle): (a,b) 50 nm, (c,d) 500 nm, (e,f) 1000 nm. Green lines correspond to *c*-domain wall.

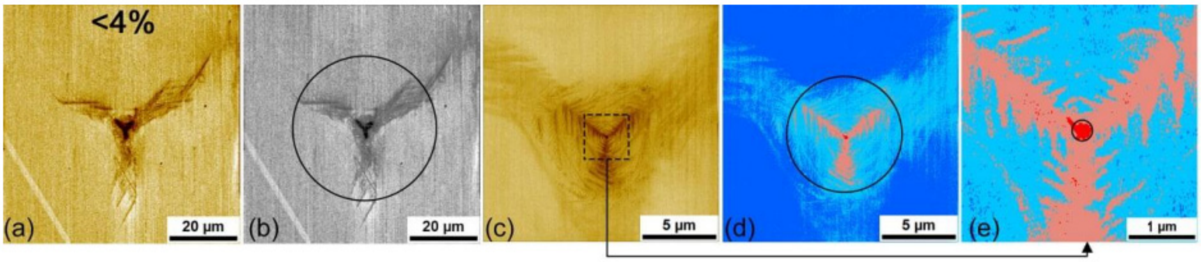

Figure S7. Schematics, illustrating, how the effective radiuses of the domain were derived in Figure 4f, in the main body of the paper (a–e).

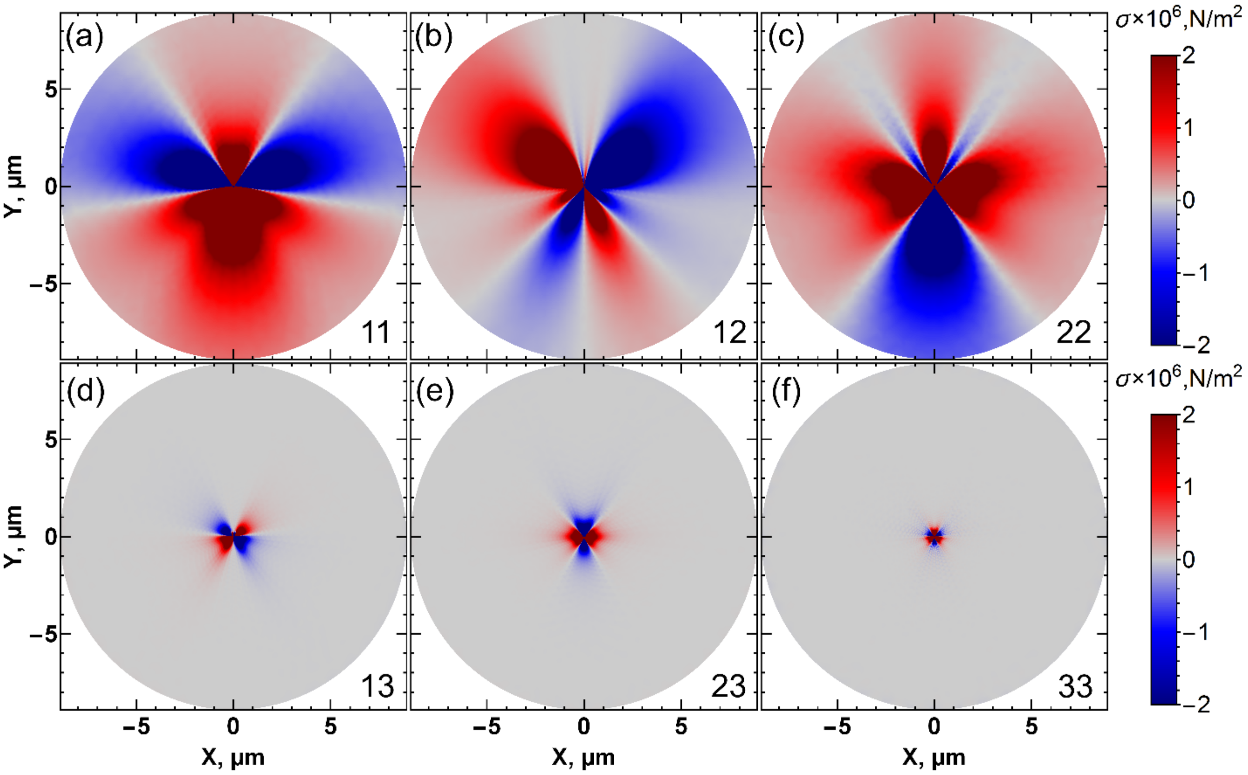

Figure S8. The distributions of stress tensor components at the 20 nm depth (a–f).

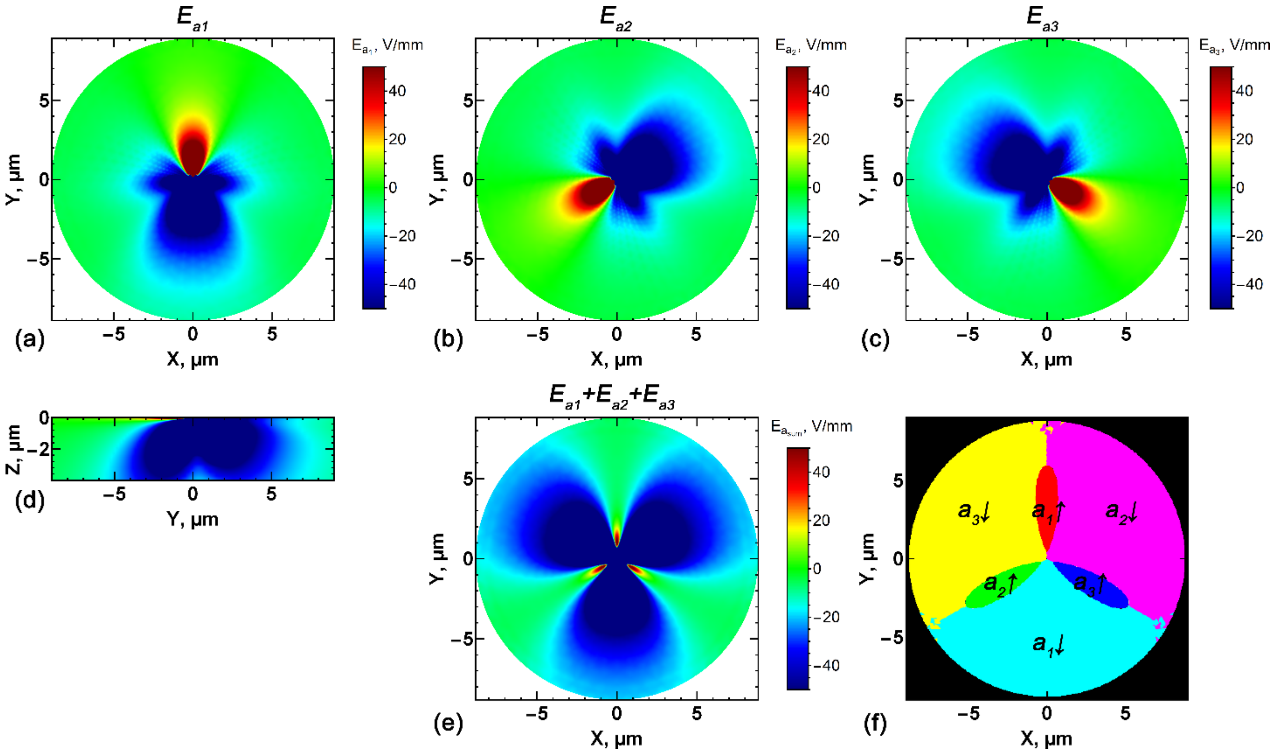

Figure S9. Simulation of the 71-degree components of electric field (along the direction of the polarization in the $a\uparrow$ domains): (a-c) XY spatial distribution of E_{a1}, E_{a2} and E_{a3} component of the electric field respectively, (d) YZ cross section of E_{a1}, (e) mathematical sum of E_{a1}, E_{a2} and E_{a3} from (a-c), (f) map illustrating the dominant directions of electric field.
